# Going virtual: mixed methods evaluation of online versus in-person learning in the NIH mixed methods research training program retreat

**DOI:** 10.1186/s12909-024-05877-2

**Published:** 2024-08-16

**Authors:** Joseph J. Gallo, Sarah M. Murray, John W. Creswell, Charles Deutsch, Timothy C. Guetterman

**Affiliations:** 1 Johns Hopkins University, Baltimore, MD USA; 2 University of Michigan, Ann Arbor, MI USA; 3 Harvard University, Boston, MA USA

**Keywords:** Research training, Mixed methods research, Research capacity building, Online education, Teaching methods

## Abstract

**Background:**

Despite the central role of mixed methods in health research, studies evaluating online methods training in the health sciences are nonexistent. The focused goal was to evaluate online training by comparing the self-rated skills of scholars who experienced an in-person retreat to scholars in an online retreat in specific domains of mixed methods research for the health sciences from 2015–2023.

**Methods:**

The authors administered a scholar Mixed Methods Skills Self-Assessment instrument based on an educational competency scale that included domains on: “research questions,” “design/approach,” “sampling,” “analysis,” and “dissemination” to participants of the Mixed Methods Research Training Program for the Health Sciences (MMRTP). Self-ratings on confidence on domains were compared before and after retreat participation within cohorts who attended in person (*n* = 73) or online (*n* = 57) as well as comparing across in-person to online cohorts. Responses to open-ended questions about experiences with the retreat were analyzed.

**Results:**

Scholars in an interactive program to improve mixed methods skills reported significantly increased confidence in ability to define or explain concepts and in ability to apply the concepts to practical problems, whether the program was attended in-person or synchronously online. Scholars in the online retreat had self-rated skill improvements as good or better than scholars who participated in person. With the possible exception of networking, scholars found the online format was associated with advantages such as accessibility and reduced burden of travel and finding childcare. No differences in difficulty of learning concepts was described.

**Conclusions:**

Keeping in mind that the retreat is only one component of the MMRTP, this study provides evidence that mixed methods training online was associated with the same increases in self-rated skills as persons attending online and can be a key component to increasing the capacity for mixed methods research in the health sciences.

**Supplementary Information:**

The online version contains supplementary material available at 10.1186/s12909-024-05877-2.

## Introduction

The coronavirus pandemic accelerated interest in distance or remote learning. While the acute nature of the pandemic has abated, changes in the way people work have largely remained, with hybrid conferences and trainings more commonly implemented now than during the pre-pandemic period. Studies of health-related online teaching have focused on medical students [1–3], health professionals [4, 5], and medical conferences [6–8] and have touted the advantages of virtual training and conferences in health education, but few studies have assessed relative growth in skills and competencies in health research methods for synchronous online vs. in-person training.

The National Institutes of Health (NIH)-funded Mixed Methods Research Training Program (MMRTP) for the Health Sciences provided training to faculty-level investigators across health disciplines from 2015–2023. The NIH is a major funder of health-related research in the United States. Its institutes span diseases and conditions (e.g., mental health, environmental health) in addition to focus areas (e.g., minority health and health disparities, nursing) and developing research capacity. Scholars in the MMRTP seek to develop skills in mixed methods research through participation in a summer retreat followed by ongoing mentorship for one year from a mixed methods expert matched to the scholar to support their development of a research proposal. Webinars leading up to the retreat include didactic sessions taught by the same faculty each year, and the retreat itself contains multiple interactive small group sessions in which each scholar presents their project and receives feedback on their grant proposal. Due to pandemic restrictions on gatherings and travel, in 2020 the MMRTP retained all components of the program but transitioned the in-person retreat to a synchronous online retreat.

The number of NIH agencies funding mixed methods research increased from 23 in 1997–2008 to 36 in 2009–2014 [9]. The usefulness of mixed methods research aligns with several Institutes’ strategic priories, including improving health equity, enhancing feasibility, acceptability, and sustainability of interventions, and addressing patient-centeredness. However, there is a tension between growing interest in mixed methods for health sciences research and a lack of training for investigators to acquire mixed methods research skills. Mixed methods research is not routinely taught in doctoral programs, institutional grant-writing programs, nor research training that academic physicians receive. The relative lack of researchers trained in mixed methods research necessitates ongoing research capacity building and mentorship [10]. Online teaching has the potential to meet growing demand for training and mentoring in mixed methods, as evidenced by the growth of online offerings by the Mixed Methods International Research Association [11]. Yet, the nature of skills and attitudes required for doing mixed methods research, such as integration of quantitative and qualitative data collection, analysis, and epistemologies, may make this type of training difficult to adapt to an online format without compromising its effectiveness.

Few studies have attempted to evaluate mixed methods training [12–15] and none appear to have evaluated online trainings in mixed methods research. Our goal was to evaluate our online MMRTP by comparing the self-rated skills of scholars who experienced an in-person retreat to an online retreat across specific domains. While the MMRTP retreat is only one component of the program, assessment before and after the retreat among persons who experienced the synchronous retreat online compared to in-person provides an indication of the effectiveness of online instruction in mixed methods for specific domains critical to the design of research in health services. We hypothesized that scholars who attended the retreat online would exhibit improvements in self-rated skills comparable to scholars who attended in person.

## Methods

### Participants

Five cohorts with a total of 73 scholars participated in the MMRTP in person (2015–2019), while four cohorts with a total of 57 scholars participated online (2020–2023). Scholars are faculty-level researchers in the health sciences in the United States. The scholars are from a variety of disciplines in the health sciences; namely, pediatrics, psychiatry, general medicine, oncology, nursing, human development, music therapy, nutrition, psychology, and social work.

### The mixed methods research training program

Formal program activities include two webinars leading up to a retreat followed by ongoing mentorship support. The mixed methods content taught in webinars and the retreat is informed by a widely used textbook by Creswell and Plano Clark [18] in addition to readings on methodological topics and the practice of mixed methods. The webinars introduce mixed methods research and integration concepts, with the goal of imparting foundational knowledge and ensuring a common language. Specifically, the first webinar introduces mixed methods concepts, research designs, scientific rigor, and becoming a resource at one’s institution, while the second focuses on strategies for the integration of qualitative and quantitative research. Retreats provide an active workshop blending lectures, one-on-one meetings, and interactive faculty-led small workgroups. In addition to scholars, core program faculty who serve as investigators and mentors for the MMRTP, supplemented with consultants and former scholars, lead the retreat. The retreat has covered the state-of-the-art topics within the context of mixed methods research: rationale for use of mixed methods, procedural diagrams, study aims, use of theory, integration strategies, sampling strategies, implementation science, randomized trials, ethics, manuscript and proposal writing, and becoming a resource at one’s home institution. In addition to lectures, the retreat includes multiple interactive small group sessions in which each scholar presents their project and receives feedback on their grant proposal and is expected to make revisions based on feedback and lectures.

Scholars are matched for one year with a mentor based on the Scholar’s needs, career level, and area of health research from a national list of affiliated experienced mixed methods investigators with demonstrated success in obtaining independent funding for research related to the health sciences and a track record and commitment to mentoring. The purpose of this arrangement is to provide different perspectives on mixed methods design while also providing specific feedback on the scholar's research proposal, reviewing new ideas, and together developing a strategy and timeline for submission.

From 2015–2019 (in-person cohorts) the retreat was held over 3 days at the Johns Hopkins University Bloomberg School of Public Health (in 2016 Harvard Catalyst, the Harvard Clinical and Translational Science Center, hosted the retreat at Harvard Medical School). Due to pandemic restrictions, from 2020–2023 the retreat activities were conducted via Zoom with the same number of lecture sessions (over 3 days in 2020 and 4 days thereafter). We made adaptations for the online retreat based on continuous feedback from attendees. We had to rapidly transition to online in 2020 with the same structure as in person, but feedback from scholars led us to extend the retreat to 4 days online from 2021–2023. The extra day allowed for more breaks from Zoom sessions with time for scholars to consider feedback from small groups and to have one-on-one meetings with mentors. Discussion during interactive presentations was encouraged and facilitated by using breakout rooms at breaks mid-presentation. Online resources were available to participants through CoursePlus, the teaching and learning platform used for courses at the Johns Hopkins Bloomberg School of Public Health, hosting publications, presentation materials, recordings of lectures, sharing proposals, email, and discussion boards that scholars have access to before, during, and after the retreat.

### Measurement strategy

Before and after the retreat in each year, we distributed a self-administered scholar Mixed Methods Skills Self-Assessment instrument (Supplement 1) to all participating scholars [15]; we have reported results from this pre-post assessment for the first two cohorts [14]. The Mixed Methods Skills Self-Assessment instrument has been previously used and has established reliability for the total items (α = 0.95) and evidence of criterion-related validity between experiences and ability ratings [15]. In each year, the pre-assessment is completed upon entry to the program, approximately four months prior to the retreat, and the post-assessment is administered two weeks after the retreat. The instrument consists of three sections: 1) professional experiences with mixed methods, including background, software, and resource familiarity; 2) a quantitative, qualitative, and mixed methods skills self-assessment; and 3) open-ended questions focused on learning goals for the MMRTP. The skills assessment contains items for each of the following domains: “research questions,” “design/approach,” “sampling,” “analysis,” and “dissemination.” Each skill was assessed via three items drawn from an educational competency ratings scale that ask scholars to rate: [16] “My ability to define/explain,” “My ability to apply to practical problems,” and “Extent to which I need to improve my skill.” Response options were on a five-point Likert-type scale that ranged from “Not at all” (coded ‘1’) to “To a great extent” (coded ‘5’), including a mid-point [17]. We took the mean of the scholar’s item ratings over all component items within each domain (namely, “research questions,” “design/approach,” “sampling,” “analysis,” and “dissemination”).

### Open-ended questions

The baseline survey included two open-ended prompts: 1) What skills and goals are most important to you?, and 2) What would you like to learn? The post-assessment survey also included two additional open-ended questions about the retreat: 1) What aspects of the retreat were helpful?, and 2) What would you like to change about the retreat? In addition, for the online cohorts (2020–2023), we wanted to understand reactions to the online training and added three questions for this purpose: (1) In general, what did you think of the online format for the MMRTP retreat?, 2) What mixed methods concepts are easier or harder to learn virtually?, and 3) What do you think was missing from having the retreat online rather than in person?

### Data analysis

Our evaluation employed a convergent mixed methods design [18], integrating an analysis of ratings pre- and post-retreat with analysis of open-ended responses provided by scholars after the retreat. Our quantitative analysis proceeded in 3 steps. First, we analyzed item-by-item baseline ratings of the extent to which scholars thought they “need to improve skills,” stratified into two groups (5 cohorts who attended in-person and 4 cohorts who attended online). The purpose of comparing the two groups at baseline on learning needs was to assess how similar the scholars in the in-person or online groups were in self-assessment of learning needs before attending the program. Second, to examine the change in scholar ratings of ability to “define or explain a concept” and in their ability to “apply to practical problems,” from before to after the retreat, we conducted paired t-tests. The goal was to compare the ratings before and after the retreat among scholars who attended the program in person to scholars who attended online. Third, we compared post-retreat ratings among in-person cohorts to online cohorts to gauge the effectiveness of the online training. We set statistical significance at *α* < 0.05 as a guide to inference. We calculated Cohen’s *d*as a guide to the magnitude of differences [19]. SPSS Version 28 was employed for all analyses.

We analyzed qualitative data using a thematic analysis approach that consisted of reviewing all open-ended responses, conducting open coding based on the data, developing and refining a codebook, and identifying major themes [20]. We then compared the qualitative results for the in-person versus online cohorts to understand any thematic differences concerning retreat experiences and reactions.

## Results

### Background and experiences of scholars

Scholars in the in-person (*n* = 59, 81%) and online (*n* = 52, 91%) cohorts reported their primary training was quantitative rather than qualitative or mixed methods, and scholars across cohorts commonly reported at least some exposure to mixed methods research (Table 1). However, most scholars did not have previous mixed methods training with 17 (23%) and 16 (28%) of the in-person and online cohorts, respectively, having previously completed a mixed methods course. While experiences were similar across in-person vs. online cohorts, there were two areas in which the scholars reported a statistically significant difference: a larger portion of the online cohorts reported writing a mixed methods application that received funding (*n* = 35, 48% in person; *n* = 46, 81% online), and a smaller proportion of the online cohorts had given a local or institutional mixed methods presentation (*n* = 32, 44% in person; *n* = 15, 26% online).

**Table 1:** Responses to questions about professional experiences from in-person cohorts 1 to 5 (*n* = 73) and online cohorts 6 to 9 (*n* = 57) of the NIH Mixed Methods Research Training Program for the Health Sciences, 2015–2023, prior to the retreat. Last column represents comparison of mean ratings across cohorts

	**In-person cohorts (1–5)**		**Online cohorts (6–9)**		
**Professional Experiences**	**n**	**Percent ‘yes’**	**n**	**Percent ‘yes’**	χ^**2**^
I am primarily trained in qualitative research	17	23.3	10	17.5	.64
I am primarily trained in quantitative research	59	80.8	52	91.2	2.78
I am primarily trained in mixed methods	8	11.0	2	3.5	2.50
I have taken a course in mixed methods	17	23.3	16	28.1	.39
I have presented mixed methods research at a national meeting	28	38.4	13	22.8	3.58
I have published a paper using mixed methods	23	31.5	19	33.3	.05
I wrote a dissertation involving mixed methods	14	19.2	7	12.3	1.12
I mentor or advise others in mixed methods research	20	27.3	13	22.8	.36
I have reviewed mixed methods applications on an NIH study section	5	6.8	6	10.5	.56
I have reviewed mixed methods applications for a foundation or other organization	14	19.2	8	14.0	.60
I have reviewed mixed methods manuscripts as a peer reviewer for a journal	27	37.0	23	40.4	.15
I have presented mixed methods research at a local or institutional meeting	32	43.8	15	26.3	4.26*
I wrote a mixed methods application that received funding	35	47.9	46	80.7	14.62***
I wrote an application that did not receive funding	34	46.6	32	56.1	1.17
I participate in a mixed methods work group	9	12.3	8	14.0	.08

^*^p ≤ .05,

**p ≤ .01

***p ≤ .001, based on χ^**2**^ tests

### Self-identified need to improve skills in mixed methods

At baseline, scholars rated the extent to which they needed to improve specific mixed methods skills (Table 2). Overall, scholars endorsed a strong need to improve all mixed methods skills. The ratings between the in-person and online cohorts were not statistically significant for any item.

**Table 2:** Responses to questions about the need to improve skills before program participation. scholars rated the extent to which they “need to improve” skills on a scale from “not at all” (coded 1) to “to a great extent” (coded 5). Data from in-person cohorts 1 to 5 (*n* = 73) and online cohorts 6 to 9 (*n* = 57) of the NIH Mixed Methods Research Training Program for the Health Sciences, 2015–2023, prior to the retreat. The ratings between the in-person and online cohorts was not statistically significant for any item

	**In-person cohorts (1–5)**		**Online cohorts (6–9)**			
	**M**	**SD**	**M**	**SD**	**t**	**Cohen's *d***
**Research Questions**						
Formulate question & aims that link modes of inquiry	4.79	0.623	4.79	0.526	0.049	0.009
State underlying philosophical assumptions	4.58	0.848	4.67	0.787	-0.628	-0.111
Rationale for mixed methods study	4.64	0.823	4.63	0.816	0.085	0.015
**Design/Approach**						
Identifying integration points in a design	4.82	0.561	4.81	0.581	0.148	0.026
Explanatory sequential designs	4.71	0.697	4.68	0.659	0.234	0.041
Exploratory sequential designs	4.68	0.762	4.60	0.821	0.635	0.112
Convergent designs	4.74	0.553	4.72	0.620	0.198	0.035
Intervention designs	4.75	0.722	4.61	0.881	0.991	0.175
Program evaluation designs	4.60	0.829	4.54	0.908	0.385	0.068
Case studies	4.14	1.122	3.95	1.420	0.851	0.150
Threats to internal validity in mixed methods	4.74	0.624	4.70	0.566	0.358	0.063
Threats to external validity in mixed methods	4.71	0.697	4.70	0.566	0.093	0.016
Diagram of the mixed methods design	4.71	0.716	4.53	0.928	1.290	0.228
**Sampling**						
Sampling strategies that link qualitative and quantitative methods (e.g., random followed by purposive)	4.71	0.677	4.72	0.620	-0.060	-0.011
Ethical principles of consent and recruitment	3.78	1.377	4.00	1.268	-0.932	-0.165
**Data Collection**						
Strategies for concurrent data collection	4.74	0.667	4.63	0.837	0.820	0.145
Strategies for sequential data collection	4.75	0.641	4.61	0.726	1.161	0.205
**Analysis**						
Combining qualitative and quantitative data (e.g., joint matrix)	4.90	0.414	4.89	0.363	0.135	0.024
Cultural consensus analysis	4.70	0.639	4.65	0.694	0.422	0.075
Inference that links qualitative and quantitative (i.e., meta-inference)	4.82	0.452	4.82	0.468	-0.033	-0.006
**Dissemination**						
Writing results incorporating both qualitative and quantitative methods in the same report	4.88	0.439	4.77	0.627	1.119	0.198
Communicate results involving both qualitative and quantitative methods to non-academic audiences	4.70	0.639	4.61	0.861	0.643	0.114

^*^p ≤ .05, **p ≤ .01, ***p ≤ .001, based on paired t-tests

### Change in self-ratings of skills after the retreat

#### Within cohorts

For all domains, the differences in pre-post assessment scores were statistically significant for both the in-person and online cohorts in ability to define or explain concepts and to apply concepts to practical problems (left side of Table 3). In other words, on average scholars improved in both in-person and online cohorts.

**Table 3:** Change in scholar mixed methods skill ratings from before retreat participation to ratings after the retreat on their ability to “define or explain” a concept (top half of table) and their ability to “apply to practical problems” (bottom half of table) on a scale from “not at all” (coded 1) to “to a great extent” (coded 5), averaged over all component items within each domain. Data from in-person cohorts 1 to 5 (*n* = 67) and online cohorts 6 to 9 (*n* = 49) of the NIH Mixed Methods Research Training Program for the Health Sciences, 2015–2023. The far right columns compare ratings of in-person to online cohorts after the retreat (negative t means online cohorts have better ratings)

	**In-person cohorts (1–5)**						**Online cohorts (6–9)**						**Comparison across in-person and online cohorts after retreat**	
	Self-rating of ability to **define or explain** concepts													
	**Before**		**After**		**Within in-person cohorts**		**Before**		**After**		**Within online cohorts**		**In-person minus online comparison**	
**Domains**	**M**	**SD**	**M**	**SD**	**t**	***d***	**M**	**SD**	**M**	**SD**	**t**	***d***	**t**	***d***
Research Questions	3.04	0.73	4.17	0.56	11.73***	1.43	2.85	0.86	4.16	0.42	10.70***	0.86	.013	0.002
Design / Approach	2.48	0.94	4.02	0.53	13.51***	1.65	2.52	0.84	3.99	0.53	12.25***	0.84	-.11	0.02
Sampling	3.22	0.95	4.01	0.63	7.06***	0.86	3.05	0.93	4.28	0.62	8.16***	1.05	-2.12*	0.39
Data Collection	2.72	1.02	4.02	0.71	9.03***	1.10	2.80	0.90	4.34	0.68	9.74***	1.11	-2.63**	0.49
Analysis	1.67	0.65	2.87	0.78	12.26***	1.50	1.80	0.82	3.47	0.66	13.15***	0.89	-4.67***	0.87
Dissemination	2.58	0.86	3.85	0.70	10.00***	1.22	2.48	0.97	4.06	0.66	9.97***	1.11	-1.68*	0.31
	Self-rating of ability to **apply concepts to practical problems**													
**Domains**	**M**	**SD**	**M**	**SD**	**t**	***d***	**M**	**SD**	**M**	**SD**	**t**	***d***	**t**	***d***
Research Questions	2.87	0.74	3.97	0.57	11.21***	1.37	2.67	0.79	4.02	0.54	10.33***	0.91	-.65	0.12
Design / Approach	2.22	0.81	3.67	0.59	14.00***	1.71	2.32	0.75	3.74	0.53	13.50***	0.74	-1.12	0.207
Sampling	3.00	0.94	3.81	0.66	6.79***	0.83	2.93	0.82	4.21	0.58	9.40***	0.96	-3.25***	0.60
Data Collection	2.49	0.98	3.73	0.76	8.51***	1.04	2.68	0.96	4.16	0.69	9.30***	1.11	-3.35***	0.62
Analysis	1.61	0.71	2.67	0.76	10.29***	1.26	1.76	0.76	3.14	0.69	12.61***	0.77	-3.73***	0.69
Dissemination	2.43	0.87	3.49	0.76	8.34***	1.02	2.34	0.91	3.74	0.75	9.22***	1.07	-1.83	0.34

^*^p ≤ .05

**p ≤ .01

***p ≤ .001, based on paired t-tests

#### Across cohorts

Online cohorts had significantly better self-ratings after the retreat than did in-person cohorts in ability to define or explain concepts and to apply concepts to practical problems (in sampling, data collection, analysis, and dissemination) but no significant differences in research questions and design / approach (rightmost column of Table 3).

### Scholar reflections about online and in-person retreats

#### Goals of training

In comparing in-person to online cohorts, discussions of the skills that scholars wanted to improve had no discernable differences. Scholars mentioned wanting to develop skills in the foundations of mixed methods research, how to write competitive proposals for funding, the use of the terminology of mixed methods research, and integrative analysis. In addition, some scholars expressed wanting to become a resource at their own institutions and providing training and mentoring to others.

#### Small group sessions

Scholars consistently reported appreciating being able to talk through their project and gaining feedback from experts in small group sessions. Some scholars expressed a preference for afternoon small group sessions, “The small group sessions felt the most helpful, but only because we can apply what we were learning from the morning lecture sessions” (online cohort 9). How participants discussed the benefits of the small group sessions or how they used the sessions did not depend on whether they had experienced the session in person or online.

#### Tradeoffs

Online participants described a tradeoff between the accessibility of a virtual retreat versus advantages of in-person training. One participant explained, “I liked the online format, as I do not have reliable childcare” (online cohort 8). Many of the scholars felt that there was an aspect of networking missing when the retreat was held fully online. As one scholar described, when learning online they, “miss getting to know the other fellows and forming lasting connections” (online cohort 9). However, an equal number of others reported that having a virtual retreat meant less hassle; for instance, they were able to join from their preferred location and did not have to travel. Some individuals specifically described the tradeoff of fewer networking opportunities for ease of attendance. One scholar wrote, being online “certainly loses some of the perks of in person connection building but made it equitable to attend” (online cohort 8).

#### Learning online

No clear difference in ease of learning concepts was described. A scholar explained: “Learning most concepts is essentially the same virtually versus in person” (online cohort 8). However, scholars described some concepts as easier to learn in one modality versus the other, for example, simpler concepts being more suited to learning virtually while complex concepts were better suited to in-person learning. There was notable variation though in the topics which scholars considered to be simple versus complex. For instance, one scholar noted that “I suppose developing the joint displays were a bit tougher virtually since you were not literally elbow to elbow” (online cohort 7) while another explained, “joint displays lend themselves to the zoom format” (online cohort 8).

#### Integrating survey responses and scholar reflections

In-person and online cohorts were comparable in professional experiences and ratings of the need to improve skills before attending the retreat, sharpening the focus on differences in self-rated skills associated with attendance online compared to in person. If anything, online attendees rated skills as good or better than in-person attendees. Open-ended questions revealed that, for the most part, scholar reflections on learning were similar across in-person and online cohorts. Whether learning the concept of “mixed methods integration” was more difficult online was a source of disagreement. Online attendance was associated with numerous advantages, and small group sessions were valued, regardless of format. Taken together, the evidence from nine cohorts shows that the online retreat was acceptable and as effective in improving self-rated skills as meeting in person.

## Discussion

Mixed methods have become indispensable to health services research from intervention development and testing [21] to implementation science [22–24]. We found that scholars participating in an interactive program to improve mixed methods skills reported significantly increased confidence in their ability to define or explain concepts and in their ability to apply the concepts to practical problems, whether the program was attended in-person or synchronously online. Scholars who participated in the online retreat had self-rated skill improvements as good or better than scholars who participated in person, and these improvements were relatively large as indicated by the Cohen’s *d* estimates. The online retreat appeared to be effective in increasing confidence in the use of mixed methods research in the health sciences and was acceptable to scholars. Our study deserves attention because the national need is so great for investigators with training in mixed methods to address complex behavioral health problems, community- and patient-centered research, and implementation research. No program has been evaluated as we have done here.

Aside from having written a funded mixed methods proposal, the online compared to earlier in person cohorts were comparable in experiences and need to improve specific skills. Within each cohort, scholars reported significant gains in self-rated skills on their ability to “define or explain” a concept and on their ability to “apply to practical problems” in domains essential to mixed methods research. However, consistent with our hypothesis that online training would be as effective as in person we found that online scholars reported better improvement in self-ratings in ability to define or explain concepts and to apply concepts to practical problems in sampling, data collection, analysis, and dissemination but no significant differences in research questions and design / approach. Better ratings in online cohorts could reflect differences in experience with mixed methods, secular changes in knowledge and availability of resources in mixed methods, and maturation of the program facilitated by continued modifications based on feedback from scholars and participating faculty [13–15].

Ratings related to the “analysis” domain, which includes the central concept of mixed methods integration, deserve notice since scholars rated this skill well below other domains at baseline. While both in-person and online cohorts improved after the retreat, and online cohorts improved substantially more than in-person cohorts, ratings for analysis after the retreat remained lower than for other domains. Scholars consistently have mentioned integration as a difficult concept, and our analysis here is limited to the retreat alone. Continued mentoring one year after the retreat and work on their proposal is built in to the MMRTP to enhance understanding of integration.

Several reviews point out the advantages of online training including savings in time, money, and greenhouse emissions [1, 7, 8]. Online conferences may increase the reach of training to international audiences, improve the diversity of speakers and attendees, facilitate attendance of persons with disabilities, and ease the burden of finding childcare [1, 8, 25]. Online training in health also appears to be effective [2, 4, 5, 25], though studies are limited because often no skills were evaluated, no comparison groups were used, the response rate was low, or the sample size was small [1, 6]. With the possible exception of networking, scholars found the online format was associated with advantages, including saving travel, maintaining work-family balance, and learning effectively. As scholars did discuss perceived increase in difficulty networking, deliberate effort needs to be directed at enhancing collaborations and mentorship [8]. The MMRTP was designed with components to facilitate networking during and beyond the retreat (e.g., small group sessions, one-on-one meetings, working with a consultant on a specific proposal).

Limitations of our study should be considered. First, the retreat was only one of several components of a mentoring program for faculty in the health sciences. Second, in-person and online cohorts represent different time periods spanning 9 years during which mixed methods applications to NIH and other funders have been increasing [9]. Third, the pre- and post-evaluations of ability to explain or define concepts, or to apply the concepts to practical problems, were based on self-report. Nevertheless, the pre-post retreat survey on self-rated skills uses a skills self-assessment form we developed [15], drawing from educational theory related to the epistemology of knowledge [26, 27].

Despite the central role of mixed methods in health research, studies evaluating online methods training in the health sciences are nonexistent. Our study provides evidence that mixed methods training online was associated with the same increases in self-rated skills as persons attending online and can be a key component to increasing the capacity for mixed methods research in the health sciences.

### Supplementary Information

Supplementary Material 1

## Data Availability

The datasets used and analysed during the current study are available from the corresponding author on reasonable request.

## Abbreviation

MMRTP: Mixed Methods Research Training Program
