# Supplementary material for: Going virtual: mixed methods evaluation of online versus in-person learning in the NIH mixed methods research training program retreat
Source: BMC Med Educ. 2024 Aug 16;24:882. doi: 10.1186/s12909-024-05877-2 (PMC11328416; doi:10.1186/s12909-024-05877-2)
Supplement: Supplementary file 1 — Supplementary Material 1 [file 12909_2024_5877_MOESM1_ESM.docx]

We intend to develop and administer an instrument to self-assess methods skills in mixed methods. The Mixed Methods Research Training Program will involve activities whose purpose is to assess the progress of participants, evaluate the program, and develop measures of skills development in mixed methods. We are required by NIH to conduct an evaluation of the program, and by accepting a position as a Scholar, students are consenting to participate in this evaluation.

This skills self-assessment consists of three sections:

**Section 1** asks about your professional experiences with mixed methods;

**Section 2** asks you to judge your ability to define or explain concepts, apply knowledge, and give advice in specific domains related to quantitative methods, qualitative methods, and mixed methods, plus what you want to learn; and,

**Section 3** provides you with an opportunity to provide feedback about the skills self-assessment and your goals.

**Section 1: Your professional experiences with mixed methods**

|  | **Yes** | **No** | **Comments** |
| --- | --- | --- | --- |
| **Background in Research Methods** |  |  |  |
| I am primarily trained in qualitative research. |  |  |  |
| I am primarily trained in quantitative research. |  |  |  |
| I am primarily trained in mixed methods. |  |  |  |
| **Professional Experiences in Mixed Methods** |  |  |  |
| I wrote a mixed methods application that received funding. |  |  |  |
| I wrote an application that did not receive funding |  |  |  |
| I participate in a mixed methods work group. |  |  |  |
| I have presented mixed methods research at a local or institutional meeting. |  |  |  |
| I have presented mixed methods research at a national meeting. |  |  |  |
| I have taken a course in mixed methods. |  |  |  |
| I have published a paper using mixed methods. |  |  |  |
| I wrote a dissertation involving mixed methods. |  |  |  |
| I mentor or advise others in mixed methods research. |  |  |  |
| I have reviewed mixed methods applications on an NIH study section. |  |  |  |
| I have reviewed mixed methods applications for a foundation or other organization. |  |  |  |
| I have reviewed mixed methods manuscripts as a peer reviewer for a journal. |  |  |  |
|  |  |  |  |

|  | **Not at all familiar** | **Familiar** | **Experienced** |
| --- | --- | --- | --- |
| **Please rate your familiarity with the following software . . .** |  |  |  |
| SPSS |  |  |  |
| SAS |  |  |  |
| STATA |  |  |  |
| R |  |  |  |
| MPlus |  |  |  |
| NVIVO |  |  |  |
| MaxQDA |  |  |  |
| Atlas |  |  |  |
| HyperRESEARCH |  |  |  |
| dedoose |  |  |  |
| **List other software you use below, if you like . . .** |  |  |  |
|  |  |  |  |
|  |  |  |  |
|  |  |  |  |
|  | **Not at all familiar** | **Read**  **some** | **Used**  **or cited** |
| **Please rate your familiarity with the following resources . . .** |  |  |  |
| The NIH *Best Practices for Mixed Methods in the Health Sciences* |  |  |  |
| *A Concise Introduction to Mixed Methods Research*  (Creswell, 2014) |  |  |  |
| *Designing and Conducting Mixed Methods Research*  (Creswell & Plano Clark, 2010) |  |  |  |
| *SAGE Handbook of Mixed Methods in Social & Behavioral Research* (Tashakkori & Teddlie, 2010) |  |  |  |
| *Mixed Methods in Health Sciences Research: A Practical Primer*  (Curry & Nunez Smith, 2015) |  |  |  |
| *Mixed Methods Research for Nursing and Health Sciences*  (Andrew & Halcomb, 2009) |  |  |  |
| **List other resources you’ve used, if you like . . . :** |  |  |  |
|  |  |  |  |
|  |  |  |  |
|  |  |  |  |

**Section 2: Skills self-assessment**

This section asks you to make ratings of specific research skills along three dimensions:

(1) your ability to **define or explain** the concept or procedure;

(2) your ability to **apply the concept or procedure to practical problems** in your discipline; and,

(3) your evaluation of what **your learning needs** are in the next year.

| **QUANTITATIVE** |  |  | |  | |  | |
| --- | --- | --- | --- | --- | --- | --- | --- |
|  |  | **My ability to**  **define / explain** | | **My ability to**  **apply to practical problems** | | **Extent to which I need to improve my skill** | |
|  |  | Not  at all  (1) | To a  great  extent  (5) | Not  at all  (1) | To a  great  extent  (5) | Not  at all  (1) | To a  great  extent  (5) |
| **Research question** |  |  | |  | |  | |
| Formulate question, aims & hypotheses | | 1 2 3 4 5 | | 1 2 3 4 5 | | 1 2 3 4 5 | |
| State the assumptions of statistical procedures | | 1 2 3 4 5 | | 1 2 3 4 5 | | 1 2 3 4 5 | |
| Use of theory or conceptual framework to be tested | |  | |  | |  | |
| **Design / approach** |  |  | |  | |  | |
| Designs involving randomization (e.g., trials) | | 1 2 3 4 5 | | 1 2 3 4 5 | | 1 2 3 4 5 | |
| Observational studies (e.g., cohort, case-control) | | 1 2 3 4 5 | | 1 2 3 4 5 | | 1 2 3 4 5 | |
| **Sampling** |  |  | |  | |  | |
| Sampling strategies (e.g., random sampling) | | 1 2 3 4 5 | | 1 2 3 4 5 | | 1 2 3 4 5 | |
| Ethical principles of consent and recruitment | | 1 2 3 4 5 | | 1 2 3 4 5 | | 1 2 3 4 5 | |
| **Data collection** |  |  | |  | |  | |
| Principles of survey methods | | 1 2 3 4 5 | | 1 2 3 4 5 | | 1 2 3 4 5 | |
| Validity of measures | | 1 2 3 4 5 | | 1 2 3 4 5 | | 1 2 3 4 5 | |
| Reliability of measures | | 1 2 3 4 5 | | 1 2 3 4 5 | | 1 2 3 4 5 | |
| Threats to internal validity of the study | | 1 2 3 4 5 | | 1 2 3 4 5 | | 1 2 3 4 5 | |
| Threats to external validity of the study | | 1 2 3 4 5 | | 1 2 3 4 5 | | 1 2 3 4 5 | |
| **Analysis** |  |  | |  | |  | |
| Descriptive statistics (e.g., comparing means) | | 1 2 3 4 5 | | 1 2 3 4 5 | | 1 2 3 4 5 | |
| Linear regression | | 1 2 3 4 5 | | 1 2 3 4 5 | | 1 2 3 4 5 | |
| Logistic regression | | 1 2 3 4 5 | | 1 2 3 4 5 | | 1 2 3 4 5 | |
| Survival analysis | | 1 2 3 4 5 | | 1 2 3 4 5 | | 1 2 3 4 5 | |
| Factor analysis | | 1 2 3 4 5 | | 1 2 3 4 5 | | 1 2 3 4 5 | |
| Structural equation models | | 1 2 3 4 5 | | 1 2 3 4 5 | | 1 2 3 4 5 | |
| **Dissemination** |  |  | |  | |  | |
| Writing results involving quantitative methods | | 1 2 3 4 5 | | 1 2 3 4 5 | | 1 2 3 4 5 | |
| Communicate quantitative results  to non-academic audiences | | 1 2 3 4 5 | | 1 2 3 4 5 | | 1 2 3 4 5 | |
|  |  |  | |  | |  | |
| **QUALITATIVE** |  |  | |  | |  | |
|  |  | **My ability to**  **define / explain** | | **My ability to**  **apply to practical problems** | | **Extent to which I need to improve my skill** | |
|  |  | Not  at all  (1) | To a  great  extent  (5) | Not  at all  (1) | To a  great  extent  (5) | Not  at all  (1) | To a  great  extent  (5) |
| **Research question** |  |  | |  | |  | |
| Formulate question & aims | | 1 2 3 4 5 | | 1 2 3 4 5 | | 1 2 3 4 5 | |
| State underlying philosophical assumptions | | 1 2 3 4 5 | | 1 2 3 4 5 | | 1 2 3 4 5 | |
| Use of theory or conceptual framework to shape the question | |  | |  | |  | |
| **Design / approach** |  |  | |  | |  | |
| Grounded theory | | 1 2 3 4 5 | | 1 2 3 4 5 | | 1 2 3 4 5 | |
| Narrative analysis | | 1 2 3 4 5 | | 1 2 3 4 5 | | 1 2 3 4 5 | |
| Phenomenology | | 1 2 3 4 5 | | 1 2 3 4 5 | | 1 2 3 4 5 | |
| Case study | | 1 2 3 4 5 | | 1 2 3 4 5 | | 1 2 3 4 5 | |
| Ethnography | | 1 2 3 4 5 | | 1 2 3 4 5 | | 1 2 3 4 5 | |
| Community based participatory research | |  | |  | |  | |
| **Sampling** |  |  | |  | |  | |
| Sampling strategies (e.g., purposive) | | 1 2 3 4 5 | | 1 2 3 4 5 | | 1 2 3 4 5 | |
| Ethical principles of consent and recruitment | | 1 2 3 4 5 | | 1 2 3 4 5 | | 1 2 3 4 5 | |
| **Data collection** |  |  | |  | |  | |
| Principles of non-directive interviewing | | 1 2 3 4 5 | | 1 2 3 4 5 | | 1 2 3 4 5 | |
| Focus group | | 1 2 3 4 5 | | 1 2 3 4 5 | | 1 2 3 4 5 | |
| Semi-structured interviews | | 1 2 3 4 5 | | 1 2 3 4 5 | | 1 2 3 4 5 | |
| Participant observation | | 1 2 3 4 5 | | 1 2 3 4 5 | | 1 2 3 4 5 | |
| Documentary materials | | 1 2 3 4 5 | | 1 2 3 4 5 | | 1 2 3 4 5 | |
| Audio-visual materials | | 1 2 3 4 5 | | 1 2 3 4 5 | | 1 2 3 4 5 | |
| **Analysis** |  |  | |  | |  | |
| Broad and fine coding of data | | 1 2 3 4 5 | | 1 2 3 4 5 | | 1 2 3 4 5 | |
| Analysis of observational data | | 1 2 3 4 5 | | 1 2 3 4 5 | | 1 2 3 4 5 | |
| Development of themes | | 1 2 3 4 5 | | 1 2 3 4 5 | | 1 2 3 4 5 | |
| Relating themes to one another | | 1 2 3 4 5 | | 1 2 3 4 5 | | 1 2 3 4 5 | |
| **Dissemination** |  |  | |  | |  | |
| Writing results involving qualitative methods | | 1 2 3 4 5 | | 1 2 3 4 5 | | 1 2 3 4 5 | |
| Communicate qualitative results  to non-academic audiences | | 1 2 3 4 5 | | 1 2 3 4 5 | | 1 2 3 4 5 | |
|  | |  | |  | |  | |
| **MIXED METHODS** |  |  | |  | |  | |
|  |  | **My ability to**  **define / explain** | | **My ability to**  **apply to practical problems** | | **Extent to which I need to improve my skill** | |
|  |  | Not  at all  (1) | To a  great  extent  (5) | Not  at all  (1) | To a  great  extent  (5) | Not  at all  (1) | To a  great  extent  (5) |
| **Research question** |  |  | |  | |  | |
| Formulate question & aims that link modes of inquiry | | 1 2 3 4 5 | | 1 2 3 4 5 | | 1 2 3 4 5 | |
| State underlying philosophical assumptions | | 1 2 3 4 5 | | 1 2 3 4 5 | | 1 2 3 4 5 | |
| Rationale for mixed methods study | | 1 2 3 4 5 | | 1 2 3 4 5 | | 1 2 3 4 5 | |
| **Design / approach** |  |  | |  | |  | |
| Identifying integration points in a design | | 1 2 3 4 5 | | 1 2 3 4 5 | | 1 2 3 4 5 | |
| Explanatory sequential designs | | 1 2 3 4 5 | | 1 2 3 4 5 | | 1 2 3 4 5 | |
| Exploratory sequential designs | | 1 2 3 4 5 | | 1 2 3 4 5 | | 1 2 3 4 5 | |
| Convergent parallel designs | | 1 2 3 4 5 | | 1 2 3 4 5 | | 1 2 3 4 5 | |
| Intervention designs | | 1 2 3 4 5 | | 1 2 3 4 5 | | 1 2 3 4 5 | |
| Program evaluation designs | |  | |  | |  | |
| Case studies | |  | |  | |  | |
| Threats to internal validity in mixed methods | | 1 2 3 4 5 | | 1 2 3 4 5 | | 1 2 3 4 5 | |
| Threats to external validity in mixed methods | | 1 2 3 4 5 | | 1 2 3 4 5 | | 1 2 3 4 5 | |
| Diagram of the mixed methods design | | 1 2 3 4 5 | | 1 2 3 4 5 | | 1 2 3 4 5 | |
| **Sampling** |  |  | |  | |  | |
| Sampling strategies that link qualitative and quantitative methods (e.g., random followed by purposive) | | 1 2 3 4 5 | | 1 2 3 4 5 | | 1 2 3 4 5 | |
| Ethical principles of consent and recruitment | | 1 2 3 4 5 | | 1 2 3 4 5 | | 1 2 3 4 5 | |
| **Data collection** |  |  | |  | |  | |
| Strategies for concurrent data collection | | 1 2 3 4 5 | | 1 2 3 4 5 | | 1 2 3 4 5 | |
| Strategies for sequential data collection | |  | |  | |  | |
| **Analysis** |  |  | |  | |  | |
| Combining q and q data (e.g., joint matrix) | | 1 2 3 4 5 | | 1 2 3 4 5 | | 1 2 3 4 5 | |
| Cultural consensus analysis | | 1 2 3 4 5 | | 1 2 3 4 5 | | 1 2 3 4 5 | |
| Inference that links qualitative and quantitative  (i.e., meta-inference) | | 1 2 3 4 5 | | 1 2 3 4 5 | | 1 2 3 4 5 | |
| **Dissemination** |  |  | |  | |  | |
| Writing results incorporating both qualitative and quantitative methods in the same report | | 1 2 3 4 5 | | 1 2 3 4 5 | | 1 2 3 4 5 | |
| Communicate results involving both qualitative and quantitative methods to non-academic audiences | | 1 2 3 4 5 | | 1 2 3 4 5 | | 1 2 3 4 5 | |
|  |  |  | |  | |  | |

**Section 3: Provide feedback about the skills self-assessment**

List any skills you think are important that we did not ask about:

Describe your goals for the Mixed Methods Research Training Program. What skills and goals are most important to you? What would you like to learn?
